# Retraction: Antibacterial and antibiofilm activities of silver-decorated zinc ferrite nanoparticles synthesized by a gamma irradiation-coupled sol–gel method against some pathogenic bacteria from medical operating room surfaces

**DOI:** 10.1039/d4ra90158d

**Published:** 2025-01-30

**Authors:** M. I. A. Abdel Maksoud, Gharieb S. El-Sayyad, Hanan S. El-Bastawisy, Rasha M. Fathy

**Affiliations:** a Materials Science Lab., Radiation Physics Department, National Center for Radiation Research and Technology (NCRRT), Egyptian Atomic Energy Authority (EAEA) Cairo Egypt; b Drug Microbiology Lab., Drug Radiation Research Department, National Center for Radiation Research and Technology (NCRRT), Egyptian Atomic Energy Authority (EAEA) Cairo Egypt adham_adham699@yahoo.com rashafathy82@gmail.com Gharieb.S.Elsayyad@eaea.org.eg

## Abstract

Retraction of ‘Antibacterial and antibiofilm activities of silver-decorated zinc ferrite nanoparticles synthesized by a gamma irradiation-coupled sol–gel method against some pathogenic bacteria from medical operating room surfaces’ by M. I. A. Abdel Maksoud *et al.*, *RSC Adv.*, 2021, **11**, 28361–28374, https://doi.org/10.1039/D1RA04785J.

The Royal Society of Chemistry hereby wholly retracts this *RSC Advances* article due to concerns with the reliability of the data.

In Fig. 13A and B, the insets of the images do not match the area they are indicating, and a section of the image in Fig. 13B was duplicated in another publication^1^ by the authors representing a different sample. In addition, the information provided in the collection data at the bottom of the images shows that these images have not been collected at a consistent magnification, while the scale bars suggest that they have, which affects the reliability of the data. The authors have not provided a satisfactory explanation.

Given the significance of these concerns, the Editor has lost confidence that the findings presented in this paper are reliable.

This retraction supersedes the information provided in the Expression of Concern related to this article.

The authors were informed about the retraction of the article. Gharieb S. El-Sayyad has not agreed with the decision, the other authors have not responded.

Signed: Laura Fisher, Executive Editor, *RSC Advances*

Date: 18th December 2024
